# Morphometric Characterization of an *Ex Vivo* Porcine Model of Functional Tricuspid Regurgitation

**DOI:** 10.1007/s10439-022-03080-2

**Published:** 2022-09-23

**Authors:** Eleonora Salurso, Michal Jaworek, Francesca Perico, Matteo Frigelli, Claudia Romagnoni, Monica Contino, Guido Gelpi, Gianfranco Beniamino Fiore, Riccardo Vismara

**Affiliations:** 1 Department of Electronics, Information and Bioengineering, Politecnico di Milano, Via Golgi 39, 20133 Milan, Italy; 2 ForcardioLab – Fondazione per la Ricerca in Cardiochirurgia ONLUS, Milan, Italy; 3 3D and Computer Simulation Laboratory, IRCCS Policlinico San Donato, San Donato Milanese, Italy; 4 Cardiovascular Surgery Unit, Fondazione IRCCS Cà Granda Ospedale Maggiore Policlinico di Milano, Milan, Italy

**Keywords:** Tricuspid valve, Passive beating heart, Tricuspid regurgitation, *In-vitro* model, Pathological model

## Abstract

Emerging treatments for tricuspid valve (TV) regurgitation require realistic TV pathological models for preclinical testing. The aim of this work was to investigate structural features of fresh and defrosted porcine right-heart samples as models of mild and severe functional tricuspid regurgitation (FTR) condition in *ex-vivo* pulsatile flow platform. Ten fresh hearts were tested *ex-vivo* under steady and pulsatile flow in typical right-heart loading conditions. Hemodynamics and 3D echocardiographic imaging of TV and right ventricle (RV) were acquired. Hearts were then kept frozen for 14 days, defrosted, and tested again with the same protocol. Morphometric parameters of TV and RV were derived from 3D reconstructions based on echo data. Fresh samples showed a slightly dilated TV morphology, with coaptation gaps among the leaflets. Sample freezing induced worsening of TV insufficiency, with significant (*p* < 0.05) increases in annulus size (annulus area and perimeter 7.7–3.1% respectively) and dilation of RV (9.5%), which led to an increase in tenting volume (123.7%). These morphologic alterations reflected into a significant increment of regurgitation fraction (27%). Together, such results suggest that fresh porcine heart samples may be a reliable *ex-vivo* model of mild FTR condition, which can be enhanced through freezing/thawing treatment to model a severe pathological condition.

**Supplementary Information:**

The online version contains supplementary material available at 10.1007/s10439-022-03080-2.

## Introduction

Functional tricuspid regurgitation (FTR) is caused by tricuspid annulus (TA) and/or papillary muscles (PM) displacement due to right ventricle (RV) dilation. This condition leads to tricuspid valve (TV) leaflets malcoaptation and tethering, and in turn to blood leakage across the valve.^6^ Moderate to severe FTR affects approximately 1.6 million patients in the United States^19^ and the prevalence increases with age. Severe FTR was found to be a predictor of long-term survival and was associated with 36% 1-year mortality.^1^ Surgical open-heart TV repair is performed only in carefully selected patients. The procedure is indeed challenging and was associated with high in-hospital mortality rates (9–18%).^4,12^ Thus, the majority of the patients is left untreated. A potential solution to these vulnerable patients can be represented by transcatheter devices. Recent advancements in the field of transcatheter technologies led to the development of novel TV transcatheter therapies.^22^ The initial clinical experiences showed safety in patients with high surgical risk^2^ and many devices are in preclinical development phase. Nevertheless, these procedures are extremely challenging for the clinicians, as the devices interact with cardiac structures and complex anatomy in beating heart conditions and the operator is guided by medical imaging only. This poses challenges not only for the final users to gain proficiency, but also for the innovators of medical industry during device development. Hence the need for platforms capable to accurately simulate the pathology in terms of hemodynamics and anatomical realism, both to provide adequate test benches for the development of novel implantable devices, and procedure simulation environments for operator training.

A key-feature of such platforms is the ability to replicate FTR main determinants. In this perspective, the most realistic approach is represented by the *in-vivo* animal models. These models provide important insights into the pathophysiology and allow evaluating the preclinical safety and efficacy of the therapies, yet they can only replicate a limited number of pathologies. Laboratory experimental cardiovascular models and numerical modelling are an alternative to *in-vivo* testing and can limit the number of animal tests. Experimental models can feature artificial polymeric^26^ or biological^16^ cardiac structures. Polymeric models offer the possibility to reproduce patient specific anatomy derived from medical imaging but the reproduction of functional atrioventricular valves is quite challenging, due to their complex anatomy.^29^ On the other hand, models exploiting biological structures provide realism in terms of anatomical features and biomechanical properties yet in some cases introduce availability and management issue. Moreover, the numerical models can provide complementary data to the experimental analysis.^23^

In our laboratory, a pulsatile-flow mock loop of the pulmonary circulation with modelled FTR was developed^9^ using defrosted porcine whole right hearts, and was successfully exploited for the *ex-vivo* hemodynamic assessment of FTR transcatheter therapies.^7,27^ Indeed, it was observed that defrosted porcine hearts, once internally pressurized to obtain pulsatile flow conditions, display TV leaflets tethering and malcoaptation, which enabled us to replicate the fluid dynamic indexes of the FTR-affected right heart with a certain realism. However, there is still a need for a detailed characterization of the structural features of the TV and RV before and after the freezing–thawing process, in order to obtain a widely usable FTR model by this methodology. Undoubtedly, with a completely characterized *ex-vivo* pathological model, the assessment of anatomical alterations secondary to the implant of a medical device can provide relevant information both in designing and in training activities. For example, the dimensioning of a novel implantable device must be based on the anatomy of the target and of the surrounding structures as well as on their alterations following the implant. These alterations, if realistically replicated in the platform, can provide insights on the execution of the devices implant procedure, enhancing the training experience.

In this work, we systematically characterized the fluid-dynamic and morphometric features of the TV-RV complex before and after the freezing/thawing process, in view of making this methodology a reliable approach to obtain well-controllable *ex-vivo* models of FTR in porcine right hearts.

## Materials and Methods

### Heart Samples Preparation

Ten fresh whole porcine hearts were collected from a local abattoir (weight: 581 ± 61 g). The left ventricles were dissected, and the inferior vena cava and coronary sinus were closed with a suture. Hydraulic connectors were secured to the right heart at the level of superior vena cava (atrial connector), pulmonary artery (pulmonary connector) and through an opening created on the septal wall (septal connector). The connectors were used to integrate the heart samples with the experimental platforms. Samples were tested first in steady flow and then in pulsatile flow experimental platforms according to a testing protocol described below. After the test, samples were disconnected from the platform and frozen for 14 days at − 18 °C keeping the hydraulic connectors in place. Samples were then defrosted at room temperature, connected to the experimental platforms, and tests were repeated.

### Experimental Platforms

#### Steady Flow Platform

**Figure 1:**
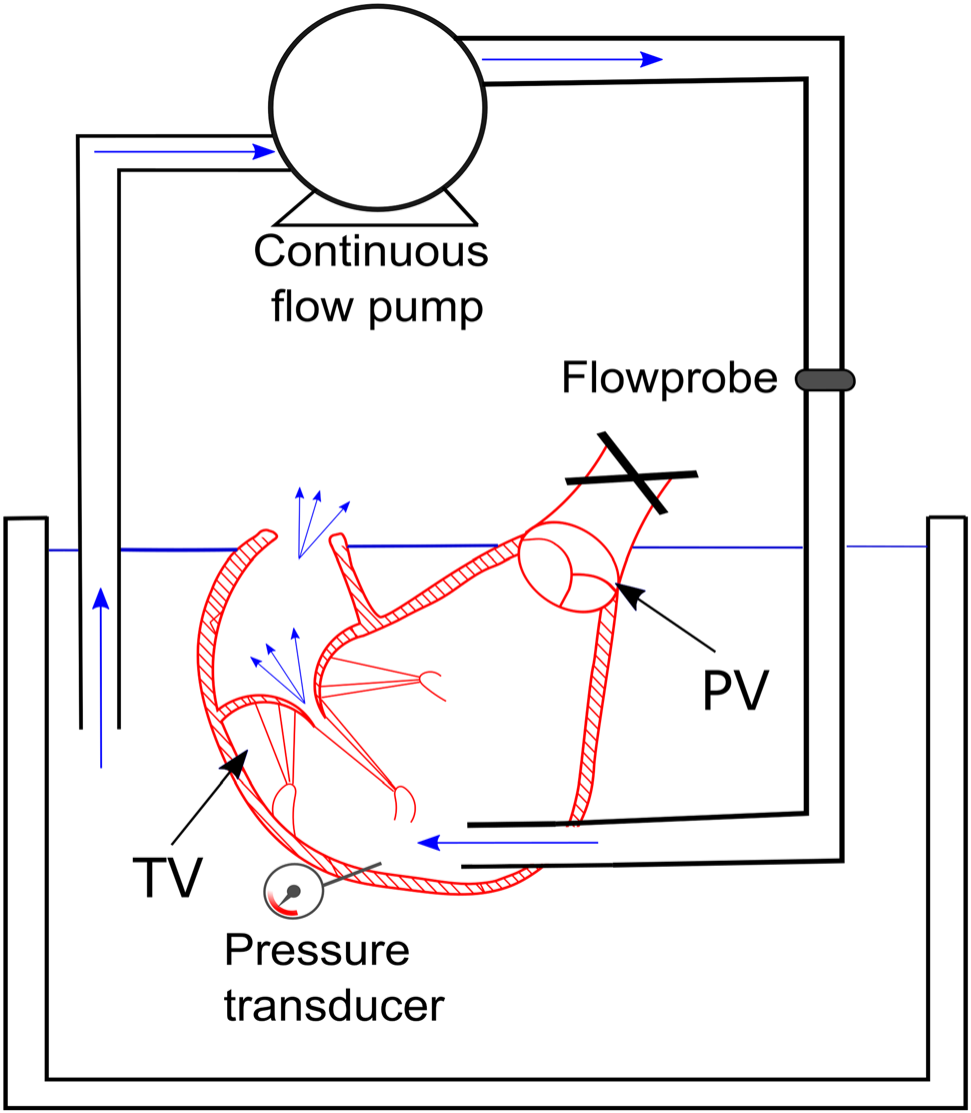
Continuous flow platform. *RV* right ventricle, *PV* pulmonary valve, *TV* tricuspid valve.

Figure 1 reports a scheme of the continuous flow platform. The pulmonary connector outflow was clamped and a centrifugal pump (Osculati Euro Pump 800) was connected to the RV, through the septal connector, inducing retrograde flow through TV. Each sample underwent steady flow characterization to assess the TV backflow in a well controllable environment. Ventricles were pressurized by adjusting the speed of the centrifugal pump, TVs were thus forced to close and tricuspid regurgitation flow rate was measured at pump outflow after reaching the steady state condition.

TV backflow was measured at ventricular pressure of 15 and 30 mmHg to represent normotensive and hypertensive systolic conditions.

#### Pulsatile Platform

**Figure 2:**
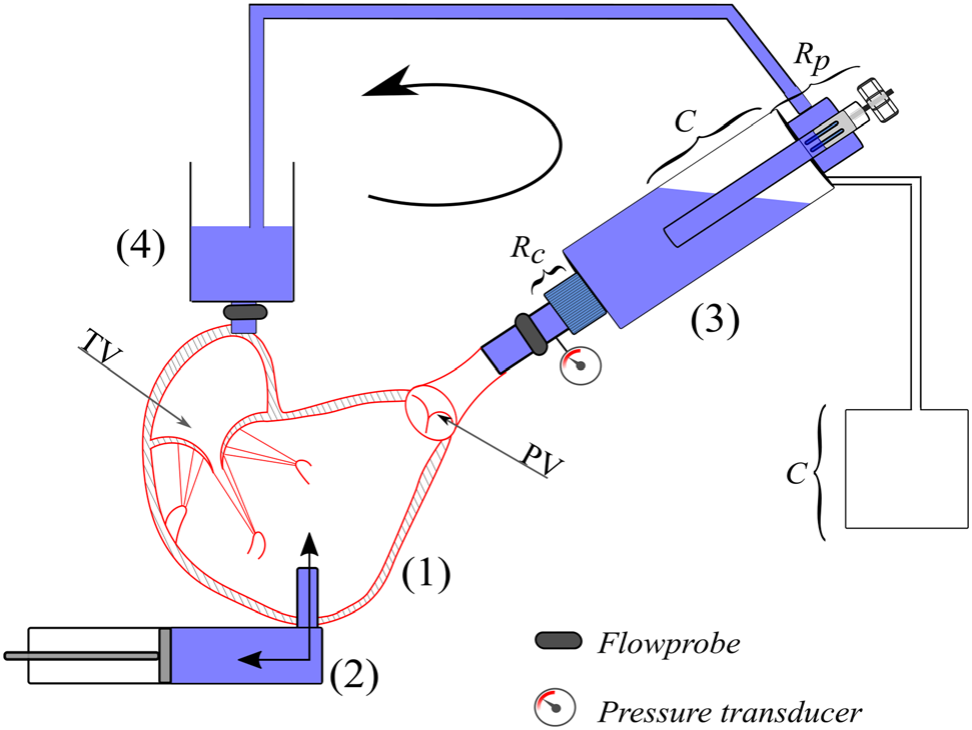
Pulsatile flow platform. (1) right ventricle; (2) pulsatile pump; (3) pulmonary impedance simulator; (4) preload reservoir; *TV* tricuspid valve, *PV* pulmonary valve.

The setup used was described in details elsewhere.^9^ The right heart was connected to the setup as shown in Fig. 2. The RV (1, Fig. 2) was connected by the septal connector to a pulsatile positive displacement pump (2, Fig. 2). The pumping system, was driven by a programmable controller which allowed to replicate the heart’s flow waveforms and to control heart rate and pump stroke volume.

During the systole, the working fluid (saline solution at room temperature) was pushed from the pump into the ventricle and through the pulmonary valve into the pulmonary impedance simulator (3, Fig. 2). The pulmonary impedance simulator is based on a three-element Windkessel model comprising a characteristic resistance (0.029 mmHg × s × mL^−1^), a compliance (16.5 mL × mmHg^−1^) and an adjustable peripheral resistance (0.22–1.18 mmHg × s × mL^−1^) which allowed for pressure regulation within a range of typical pulmonary pressure values. The outflow of the impedance simulator was connected to a reservoir (4, Fig. 2) which provided preload to the right atrium. The heart was immersed in a water tank to facilitate echocardiographic imaging. Pulmonary and tricuspid flow rates were respectively measured downstream of the pulmonary artery and upstream of the superior vena cava, by means of a transit-time flowmeter equipped with a 1″ probe (HT110R, Transonic System, Inc., Ithaca, NY, USA). Pulmonary artery pressure was measured with transducers (143PC03D model, 140PC series, Honeywell, Inc., Morristown, NJ, USA) at the level of the impedance simulator. Fiberscope imaging (OTV S7 endoscope with rigid lens (Olympus Corp., Tokyo, Japan) enabled direct visualization of the TV in the atrial view. Three-dimensional ECG-gated echocardiography of TV and RV was acquired through an iE33 ultrasound system equipped with transthoracic and transoesophageal probes X7-2t and X5-1, respectively. (Philips, Eindhoven, The Netherlands). The transoesophageal probe was positioned on the right atrium and the transthoracic probe was placed distally and oriented towards the heart apex to acquire the TV and the RV echocardiographic volumetric images, respectively. Working conditions were set as follows: heart rate 60 bpm and pump stroke volume 70 mL, which led to a mean pulmonary artery pressure (PAP) of 35.12 ± 11.9 mmHg and a Cardiac Output (CO) of 1.21 ± 0.34 L/min as representative of a hypertensive condition. The specimens showed high compliance resulting in lowered cardiac output when compared with typical values. After the system reached stable working conditions, pressure, and flow signals of 10 cardiac cycles were acquired with an A/D converter (DAQ USB 6210, National Instruments, Austin, TX, USA) at a sampling frequency of 200 Hz.

#### Data Processing

After averaging the raw data over 10 cardiac cycles, the following indexes were calculated:

- Tricuspid Regurgitant fraction (TRF) [%] calculated as the ratio between the TV backflow volume and the ventricular systolic stroke volume;
- Cardiac Output (CO) [L/min], expressed as mean value of the pulmonary flow rate;
- Pulmonary regurgitant volume (PRV) [mL], calculated as the time integral of the negative pulmonary flow rate.

**Figure 3:**
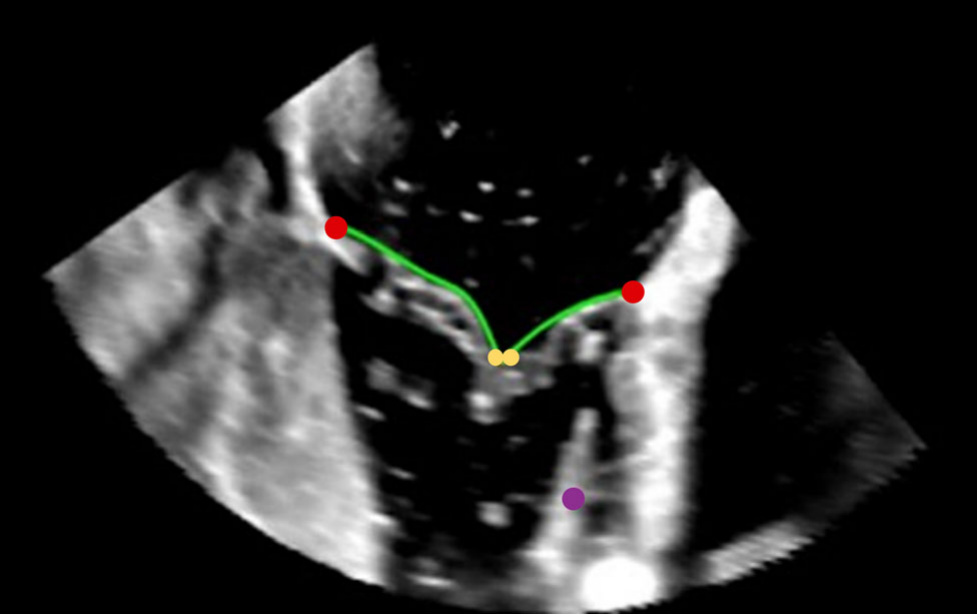
Echocardiographic imaging of the TV used for the segmentation: annulus (red points), leaflet profile (green lines), leaflet free margin (yellow dots) anterior papillary muscle (purple dot).

A dedicated software developed in Matlab® (The Mathworks, Inc., Natick, MA, US) was used to assess TV morphology from the 3D acquisitions, following an approach that was initially adopted for 4 dimensional multidetector computed tomography data.^15^ At first, the TV long axis was defined at the end-systole phase, defined as the time instant prior to valve opening. Subsequently, 18 planes evenly rotated (20degrees) around the TV axis were automatically defined by interpolating the volumetric data. On each plane, the atrial contour of the TV leaflets was manually traced (green lines in Fig. 3); two annular points (red dots in Fig. 3) and two points belonging to the free margin (yellow dots in Fig. 3) were manually identified. Additionally, on a single plane, a point belonging to the anterior papillary muscle (purple dot in Fig. 3) was selected to identify a reference anatomical landmark. Annular points were fitted through 4th order Fourier functions and the TV surface at end-systole was automatically reconstructed exploiting Delaunay triangulation.^20^ In addition, 3D tenting volume was calculated as the portion of space limited by the 3D annulus plane and the leaflets surface following the method described in Ref. 8.

**Figure 4:**
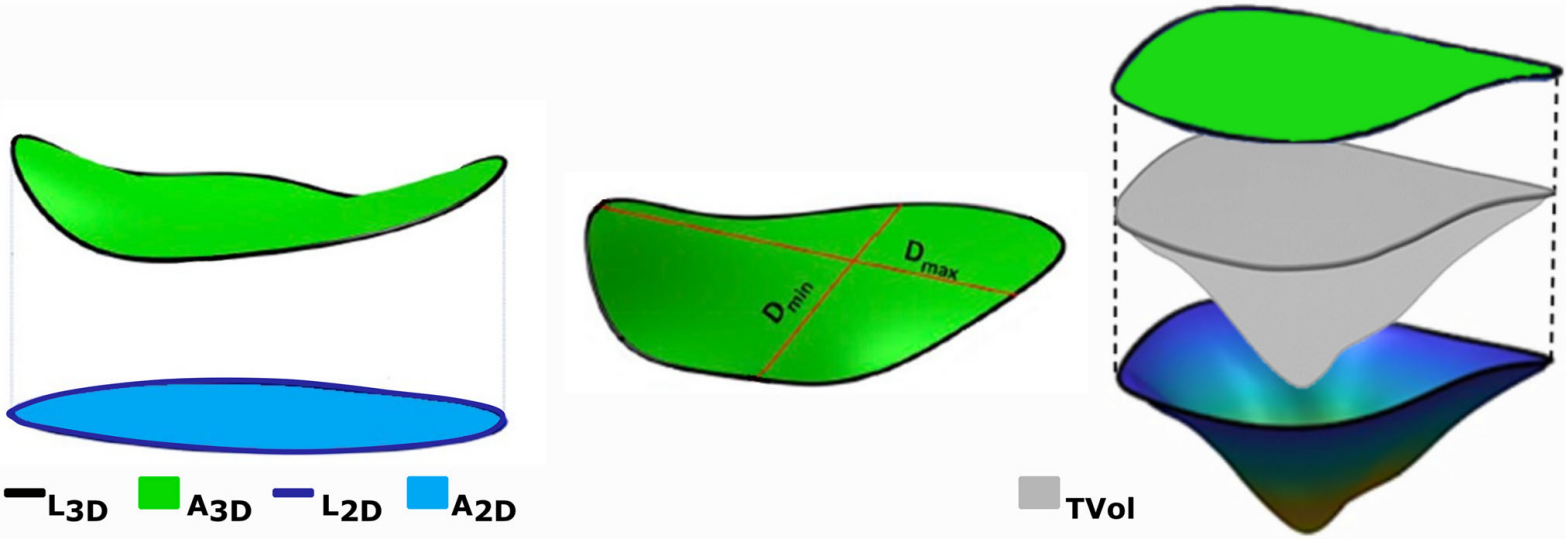
TV morphometric parameters obtained from 3D reconstruction of echocardiographic data: (a) 2D and 3D perimeter and area (L2D, L3D, A2D, A3D); (b) Maximum (*D*_max_) and minimum (*D*_min_) TV annulus diameter; (c) Tenting volume (*T*_vol_).

The following morphometric parameters were computed from the 3D reconstructions (Fig. 4):

- Bidimensional and tridimensional perimeter (L2D, L3D): perimeters defined on the bidimensional (projection of the annulus on the best-fit plane) and three-dimensional TV profile. (Fig. 4a)
- Bidimensional and three-dimensional area (A2D, A3D): areas enclosed in the bi- and three- dimensional TV profiles. (Fig. 4a)
- Maximum and minimum diameter (*D*_max_, *D*_min_): the longest and shortest chord, respectively, connecting two annular points and crossing the centroid of the TA points (Fig. 4b)
- Tenting volume (*T*_Vol_): the volume included between the three-dimensional plane of the valve anulus and the surface of the valve leaflets (Fig. 4c)

**Figure 5:**
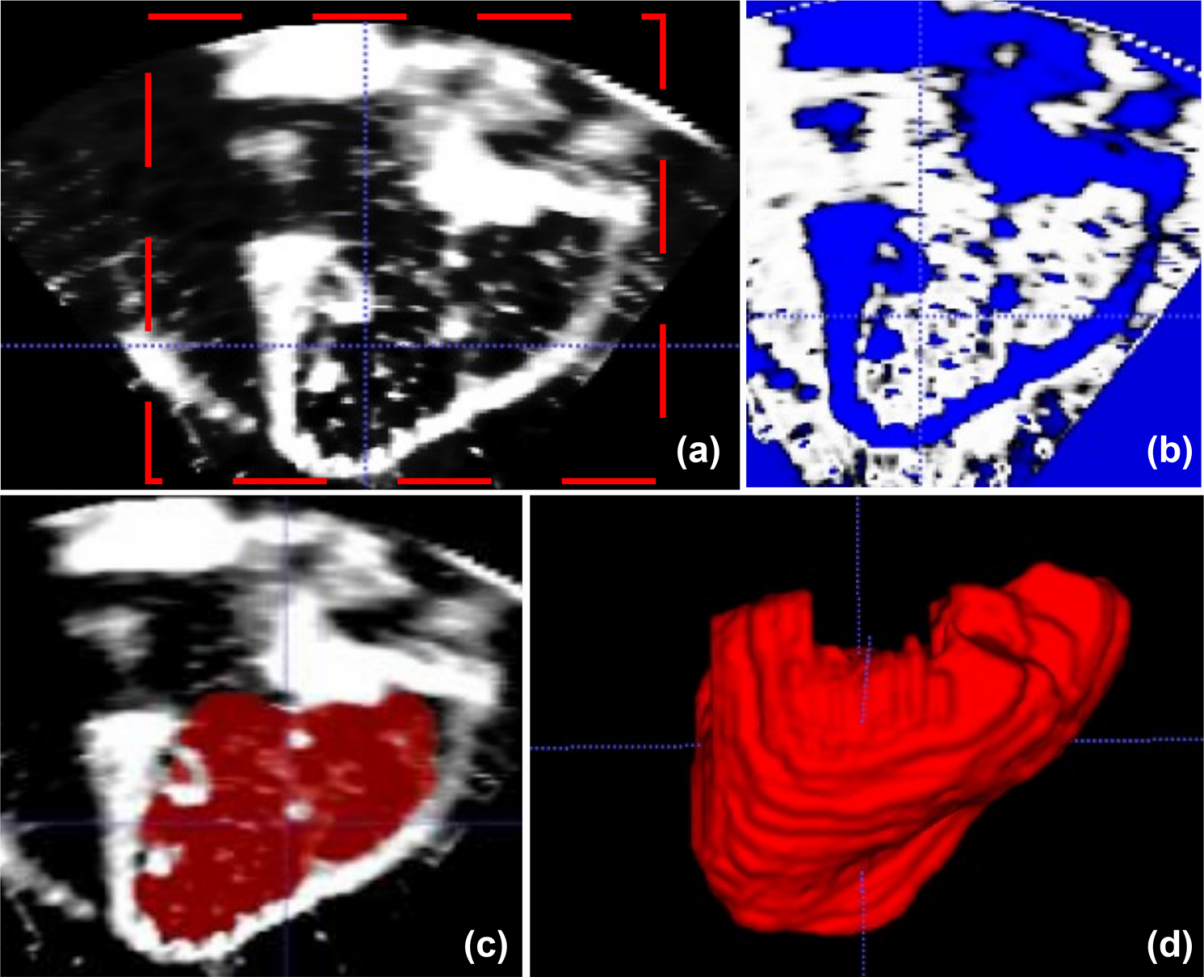
Different stages of the 3D volume reconstruction: (a) definition of the ROI for the segmentation process; (b) thresholding method; (c) end of the region growing process; (d) 3D volume which results from the segmentation process.

3D echocardiographic data of the RVs were processed offline in the open-source software ITK-SNAP (Penn Image Computing and Science Laboratory, University of Pennsylvania, Philadelphia). This software enabled the evaluation of the RV intracavitary volume recreating the 3D anatomy through a four-step semi-automatic segmentation process:

i. the region of interest (ROI) was manually defined (red dashed line in Fig. 5a), thus allowing to perform the segmentation process on a portion of the 3D dataset rather than the whole image;
ii. pre-segmentation process was performed through a thresholding algorithm. In this stage the software transformed the anatomical image in a so-called speed image, with values ranging from − 1 to 1. The aim of this stage was to define speed values close to 1 in the structures of interest. Exploiting the thresholding method, voxel intensities that were included into the user-defined upper and lower thresholds were mapped to positive speed values (white regions in Fig. 5b);
iii. RV endocardial cavity was automatically segmented (Fig. 5c) by means of region growing algorithms embedded in the ITK-SNAP software. Seeds were placed in the structure of interest and the segmentation area started to expand iteratively in the positive portions of the speed image and contracting over the negative ones. The region growing process automatically terminated when the RV endocardial border was reached; of note, it could be manually interrupted if the segmented area exceeded the structure of interest;
iv. manual refinement of the segmented area could be performed to correct for minor imperfections. This stage could be useful to air bubbles or the septal connector into the RV cavity. These structures displayed grey-scale intensity values similar to the ones of the RV endocardial border, thus displaying speed values close to − 1, which effectively excluded them from the structures of interest. The resulting volume of the RV cavity (Fig. 5d) was reconstructed and quantified in ITK-SNAP.

#### Statistical Assessment

The data were expressed as mean ± standard deviation after confirming their normal distribution (Shapiro–Wilk test). Statistical differences between parameters of fresh and defrosted samples were assessed using a paired *t*-test.

## Results

**Figure 6:**
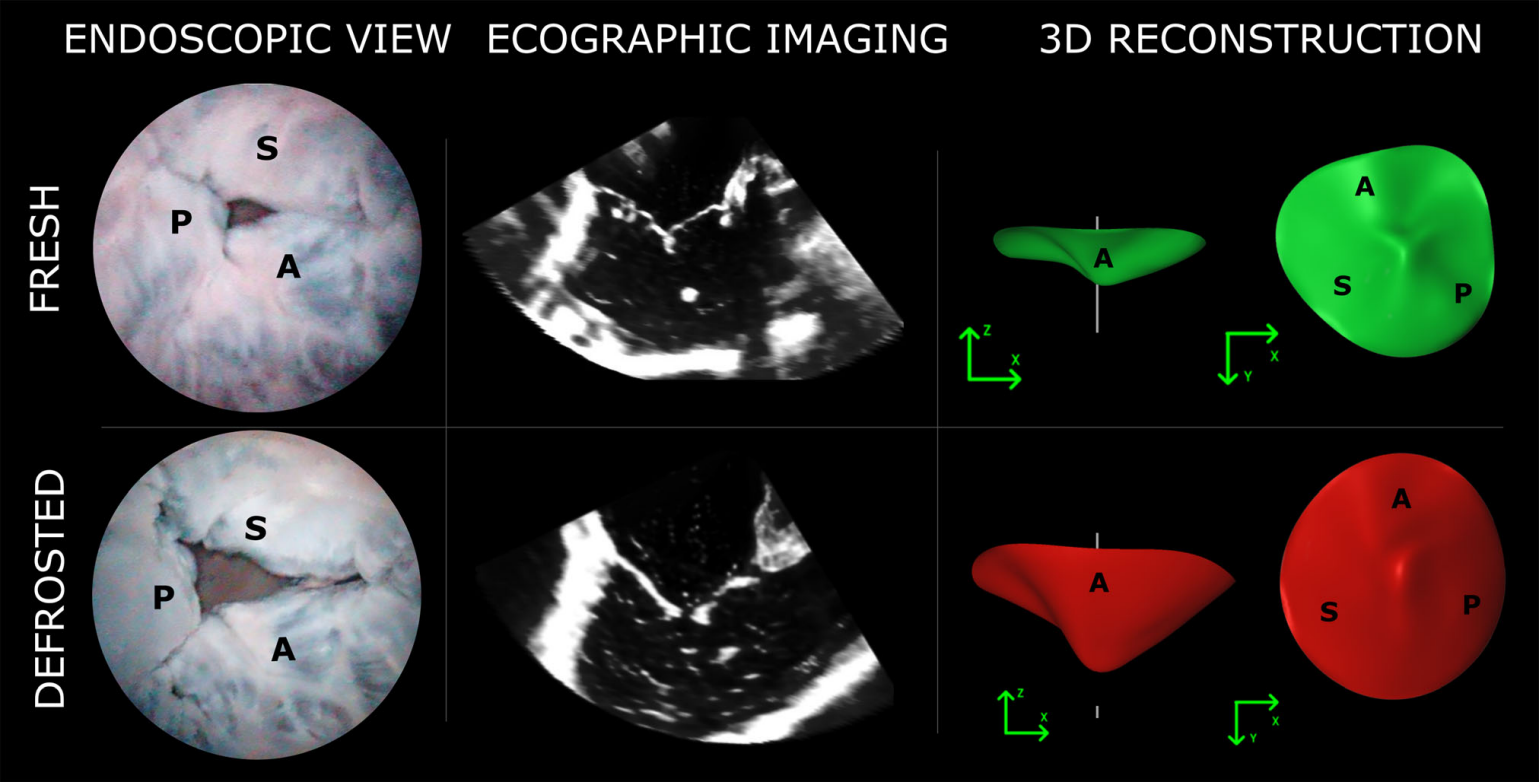
Comparison of fresh and defrosted TV in endoscopic, echographic and 3D model reconstruction of heart sample 6. A, P, S: anterior, posterior and septal leaflet.

The freezing and defrosting treatment of porcine heart samples yielded TV and RV morphological and hemodynamic alterations with respect to fresh heart samples. Qualitatively, the effects of the freezing process on TV configuration in end systole can be appreciated from the videos enclosed as supplementary materials containing exemplary fiberscopic images, 2D echocardiographic planes and TV 3D reconstructions (an example is also reported in Fig. 6): the defrosted samples showed worse coaptation of TV leaflets and more extensive leaflets tethering with respect to fresh samples.

### Fluid Dynamic Assessment

**Figure 7:**
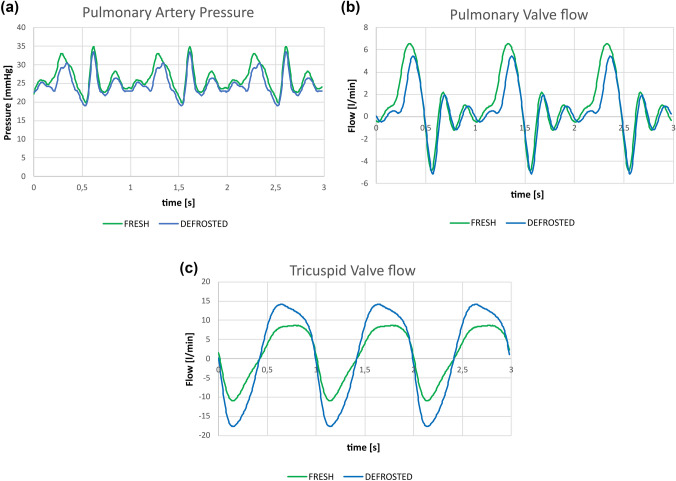
Experimentally obtained pulmonary artery pressure (a), pulmonary valve flow (b) and tricuspid flow curves (c) in fresh (green) and defrosted (blue) condition for the same heart sample.

Table 1 shows the variation of the analysed quantitative indexes in fresh and defrosted samples. Figure 7 shows an example of the raw data (pulmonary pressure, pulmonary and tricuspid flow) recorded for a sample in the fresh and defrosted conditions.

**Table 1:** Fluid dynamic and morphometric indexes analysed: BF—15 mmHg (L/min), backflow value evaluated in steady flow condition at intraventricular pressure of 15 mmHg; BF—30 mmHg (L/min), backflow value evaluated in steady flow condition at intraventricular pressure of 30 mmHg; TRF (%), tricuspid regurgitant fraction, calculated as the ratio between the backflow volume and stroke volume; CO (L/min), cardiac output expressed as mean value of the pulmonary flow rate; PRV (mL), pulmonary regurgitation volume calculated as the time integral of the negative pulmonary flow rate; L2D-L3D (mm), bidimensional and three-dimensional perimeter defined on the bidimensional (projection of the annulus on the best-fit plane) and three-dimensional TV profile respectively; A2D-A3D (mm^2^)—bidimensional and three-dimensional areas enclosed in the bi-tri dimensional TV profiles; *T*_vol_ (mL), tenting volume, volume included between the three-dimensional plane of the valve anulus and the surface of the valve leaflets; RV volume (mL), volume of the right ventricle.

	Fresh	Defrosted	Mean variation (%)	*p*-value
*Fluid dynamics*				
BF—15 mmHg (L/min)	0.4 ± 0.3	1.7 ± 0.9	551.0	0.002
BF—30 mmHg (L/min)	1 ± 0.5	3.5 ± 1.6	297.8	< 0.001
TRF (%)	67.1 ± 8.5	84.0 ± 4.0	26.9	0.01
CO (L/min)	1.7 ± 0.9	0.9 ± 0.9	53.2	0.035
PRV (mL)	7.9 ± 2.1	8.3 ± 2.6	3.9	0.539
*Morphometrics*				
*D*_max_ (mm)	50.2 ± 3.6	51.7 ± 2.4	3.3	0.04
*D*_min_ (mm)	43.1 ± 3.5	45.1 ± 3.6	4.9	0.04
L2D (mm)	149.2 ± 10.0	153.7 ± 8.1	3.1	0.02
L3D (mm)	152.8 ± 10.1	156.9 ± 7.6	2.9	0.02
A2D (mm^2^)	1735.9 ± 234.1	1861.5 ± 210.7	7.7	0.008
A3D (mm^2^)	1808.0 ± 238.0	1919.7 ± 197.9	6.7	0.009
*T*_Vol_ (mL)	6.0 ± 0.8	13.3 ± 4.5	123.7	0.001
RV volume (mL)	185.8 ± 40.8	202.0 ± 36.4	9.5	0.05

Data are reported as mean value ± standard deviation

Tests in steady flow condition showed fivefold and threefold TV backflow increase in freeze-treated samples at intraventricular pressures of 15 mmHg (*p* = 0.002) and 30 mmHg (*p* < 0.001), respectively. Pulsatile flow tests reported statistically significant variations in the TRF value which increased by 27% after freezing treatment (*p* = 0.01). This indicates alterations in RV and TV functioning as also qualitatively noticeable by the tricuspid valve flow reported in Fig. 7c. The increase in TRF directly affected the pulmonary flow as can be appreciated by the lowered positive peak of the pulmonary flow curve reported in Fig. 7b and as highlighted by a 53.2% reduction of CO in defrosted samples. On the other hand, no statistically significant changes were observed in Pulmonary regurgitation volume (PRV) values (*p* = 0.54) as confirmed by the unvaried negative portion of the curve (Fig. 7b) which indicate no direct effect of freezing treatment on the functioning of the pulmonary valve.

### Morphometric Assessment

The quantitative investigation of TV and RV morphology suggests a high impact of the freezing process on the geometry of the valve at end systole as annular and ventricular dilation were observed. Quantitatively, sample freezing induced significant (*p* < 0.05) increase in annulus size: annulus area and perimeter increased by 6.7–2.9% and 7.7–3.1% in 3D and 2D reconstructions, respectively. Similarly, Dmax and Dmin values increased by 3.3% and 4.9%, respectively. The reported annular dilation, together with RV volume increase by 9.5% (*p* = 0.05), accounted for a 124% (*p* = 0.001) increase in TV tenting volume.

## Discussion

In this work we presented an effective and simple technique to replicate the structural features of different grades of FTR with excised porcine hearts in an *ex-vivo* pulsatile flow platform. Fresh heart samples showed to be a reliable model of mild FTR condition, while defrosted ones well represented a severe pathological condition.

Fresh samples exhibit, on average, leaflet tethering and a slightly dilated morphology with coaptation gaps among the leaflets. This was directly reflected by the presence of regurgitant flow. The freezing process promoted both annular and ventricular dilation, resulting in increased tenting volume, RV volume, annulus area and annulus perimeter. The same trend of alteration can be found in the clinical echocardiographic assessment of patients with different grades of FTR.^15,21,24^ Clinically, mild FTR is characterized by lower annular and ventricular dimensions, less leaflets tenting and lower coaptation gaps with respect to severe FTR conditions. A possible explanation for this modifications may be represented by the dehydration^25^ induced by the freeze–thaw process, which could lead to modifications in the collagen matrix arrangement that, in turn, could induce changes in the mechanical properties of the tissues. This warrant further histological assessment before and after the freezing process. Of note, we observed high sample-to-sample variability when using fresh samples, with some samples showing gaps between the leaflets, others behaving close to physiological, with negligible regurgitation. The freeze–thawing process smoothed the differences observed among the samples. The treatment induced a more severe pathology, together with a higher controllability and repeatability of the experimental model.

Bench models able to reproduce the complexity of the TV apparatus constitute an important asset as they can assist, facilitate, and speed up the development of novel transcatheter techniques. Different strategies are adopted to model the FTR condition in preclinical experimental platforms. Considering *ex-vivo* models, several research groups make use of hydraulic mock loop housing excised TV.^14,17,18^ In these experimental models the pathology is replicated by design solutions aimed at altering the annulus size and papillary muscles position. Although the advantage of being a well controllable strategy, these models are complex in the setting up phase and the absence of anatomical structures surrounding the valve apparatus limits their application to selected devices. A trade-off between controllability and realism is represented by *ex-vivo* models employing whole hearts. These modes reproduce the pathology through spontaneous right heart dilation following ventricle pressurization, and have the benefit to preserve the anatomical realism without complicating the preparation phase. Several researchers made use of this model^11,30^ in steady flow condition, thus not replicating the valve dynamics during the cardiac cycle. The model developed in our lab^9^ showed to be a reliable and easily controllable strategy, with a realistic reproduction of the fluid dynamic and valve dynamics. The work presented here strengthened the model reliability by confirming the realism from the morphological point of view. The proposed model could have multiple applications. It could be exploited by medical devices developers as a valid and cost-effective tool for fast feedback on the design of their devices. It could be used as a research platform to test the feasibility of novel therapeutic concepts or, furthermore, be employed by clinicians as a training environment for novel devices implantation. In the context of clinical research, the model could also give insights on some of the clinically debated aspects of the FTR pathology and treatments. Clinical experiences show that the efficacy of the current FTR treatments depend on the underlying mechanism (annular and/or ventricular dilation) and on the stage of the pathology.^3,13^ The proposed model could be used as a tool to match the suitable therapeutic strategy to the underlying mechanism. In particular, it could be used to evaluate the efficacy of a device in case of mild RV dilation (exploiting fresh samples) or severe RV dilation (exploiting defrosted samples). Furthermore, post-operative RV dilation was observed as a determinant of pathology recurrence.^5^ Our model can simulate long-term dilation of the RV. The device or surgical technique can be tested in the mild-FTR experimental model (i.e. in fresh samples) and then in the severe-FTR experimental model (i.e. in frozen/defrosted samples). This could provide an insight on the treatment efficacy in the setting of progressive RV dilation.

Additionally, this work provides 3D anatomical data of porcine TV which could be a reference to the scientific community in the context of translating the *ex-vivo* and *in-vivo* porcine studies to human data.

### Limitations

The study carries intrinsic limitations of *ex-vivo* passive beating heart platform. The heart lacked the natural heart muscle contraction and the pumping system induced paradoxical motion of the ventricle resulting in ventricle volume increase during systole and decrease in diastole. Similar issues were also present in left ventricle mock loop^10^ and the possible influence of this paradoxical ventricular behaviour was compensated by the lack of papillary muscles contraction. This preserved an efficient functional morphology of the mitral valve preventing leaflet bulging towards the left atrium.^28^ In TV case, the same principles apply, however due to RV properties the systolic volume increase leads to exaggerated PM displacement, leaflets tethering and thus regurgitation. The transvalvular pressure drop recordings were omitted in this work. The pressure ports connected to the heart chambers would have introduced shadows in the echocardiographic images, which are the focus of this work. Previous works from our group^8,27^ already assessed the transvalvular pressure drop, showing that our experimental platform allows to obtain realistic and repeatable values. The transvalvular pressure drop is an important parameter to be considered during evaluation of the heart valve therapies and therefore in the future it would be of interest to adapt the platform to perform simultaneous acquisitions of ultrasound and pressure data.

Moreover, morphological evaluation was performed by semi-automatic 3D echocardiographic data segmentation which is an operator dependent process. To limit operator-dependency of the collected data, the segmentations were performed independently by two operators and the averaged values were reported.

In conclusion, we quantitatively characterized the 3D morphological features of TV and RV in the *ex-vivo* passive beating right heart platform and presented a methodology to model different grades of FTR severity exploiting fresh and defrosted porcine hearts. The adopted strategy allowed selective replication of mild or severe morphological and hemodynamic features of FTR, making the porcine heart model a reliable tool to increase efficacy and efficiency of FTR treatment strategies.

## Supplementary Information

Below is the link to the electronic supplementary material.

Supplementary file1 (MP4 9921 kb).

## Abbreviations

TV: Tricuspid valve
RV: Right ventricle
TA: Tricuspid annulus
PV: Pulmonary valve
FTR: Functional tricuspid regurgitation
CO: Cardiac output
PAP: Pulmonary artery pressure
TRF: Tricuspid regurgitation fraction
PRV: Pulmonary regurgitation volume
PM: Papillary muscles
L2D: Bidimensional perimeter
L3D: Tridimensional perimeter
A2D: Bidimensional area
A3D: Tridimensional area
Dmax: Maximum diameter
Dmin: Minimum diameter
TVol: Tenting volume
